# Loss of homeland: a qualitative study of the changes in perception of relocated Sichuan earthquake survivors with posttraumatic stress disorder

**DOI:** 10.1186/s12888-020-02789-5

**Published:** 2020-07-31

**Authors:** Zhengjia Ren, Junwei Guo, Chunsong Yang

**Affiliations:** 1 Department of Clinical Psychology, Southwest Hospital, The first Hospital affiliated to Army Medical University (Third Military Medical University), Chongqing, China; 2 The psychological clinic of Chongqing traditional Chinese Medicine Hospital, Chongqing, China; 3 West China Second Hospital, Sichuan University, Chengdu, China

**Keywords:** Relocation, Earthquake survivors, Posttraumatic stress disorder, Qualitative research

## Abstract

**Background:**

This research aims to explore the life experiences of relocated earthquake survivors with PTSD and develop a conceptual framework for understanding their life experiences.

**Method:**

Interviews were conducted with twenty-three participants. The participant selection, data collection and analysis were based on grounded theory methodology. A theoretical model called “loss of homeland” was developed.

**Results:**

Loss of homeland was the most important condition that influenced the relocated participants’ self-identity, social connections, and meaning system. These aspects were categorized into existential changes, lost connections, and changes in identity. Post-disaster relocation threatens individuals’ sense of meaning, integrity of self, and sense of belonging, affects every aspect of everyday life and shatters their inner and outer harmony.

**Conclusions:**

Further research guided by this theoretical model is needed to inform post-disaster mental health services and relocation policy. Mental health professionals and policy makers can make more informed decisions in terms of disaster relocation policy and manage post-disaster psychological disturbances by focusing on both places and people.

## Background

The victims of the devastating Sichuan earthquake on May 12, 2008 have yet to fully process their extreme life experience. Their psychosocial pain is still evident and has not diminished over time, even many years after the earthquake [1, 2]. Among the people who experienced the earthquake, between 10.4% (in moderately damaged areas) and 47.3% (in heavily damaged areas) were diagnosed with posttraumatic stress disorder (PTSD) [3, 4]. The victims were impacted not only by the earthquake but also by post-earthquake relocation. A previous study reports that both traumatic and posttraumatic postmigration stress contribute similarly to the expression of PTSD symptoms [5]. A previous study identified relocation as a pivotal factor associated with the physical and psychological problems after the Sichuan earthquake [6]. It has been widely reported that relocation after traumatic life incidents increases the difficulties of disaster survivors and causes psychological distress by disrupting social networks, the usual sources of medical care and pharmaceuticals, and access to social services and regular routines [7, 8]. In addition, relocation to other communities can cause cultural discontinuity and a sense of exclusion from other communities, which have been linked to high rates of depression, alcoholism, suicide, and violence [9–11]. Tuan (1977) states that places maintain a quality of permanence and act as the “centers of felt value” where our physical and psychosocial needs and security are maintained [12]. Based on the theory proposed by Anthony Giddens, “place” is conceptualized by the idea of locale, which refers to the physical settings of social activity, as geographically situated [13]. Human activities, such as social engagement, religious or memorial services, community ceremonies, oral histories of the community or family, and burials, occur in spaces, and people transform spaces into meaningful places to create a feeling of place that serves as the foundation of a sense of belonging [13, 14]. A sense of belonging is based on who a person is (identity) and from where the person originates (place) [15]. As described by Chinese sociologist Fei, attachment to “soil” reflects the inner psychological world of rural Chinese farmers and is central to their self-identity, sense of belonging, sense of meaning and value and is the root of their lives [16].

Health must be understood not only in terms of individual characteristics but also in relation to locales [17, 18]. Displacement threatens the ontological security of earthquake survivors due to the loss of a familiar physical or social environment, which alters their sense of self, place, and belonging and interferes with their reentry into healthy and meaningful societal roles [19–21]. In particular, traumatized earthquake survivors are forced to move to a new community.

The cultural meaning of relocation and how relocation leads to psychological disturbances among Sichuan earthquake survivors have not been addressed in the academic literature. A qualitative, survivor-centered approach could provide a deep understanding “beyond measures and numbers” and is an important complement of quantitative research [22].

Our current study attempts to explore relocated survivors’ perspectives of relevant processes and contexts and the interplay among relocation, mental health vulnerability, and the cultural expression of symptoms among Sichuan earthquake survivors; this approach may offer a deeper understanding and valuable information that advances theory building and culturally sensitive trauma-informed care.

## Methods

This study aims to explore the life experiences of relocated earthquake survivors with PTSD. A grounded study design, which is a suitable method when new areas are investigated in an exploratory manner, was adopted.

### Participant selection

The eligible participants were earthquake survivors who screened positive for PTSD and were relocated to other communities. The participants had previously lived in villages, many of which were destroyed by the earthquake. Entire communities were destroyed by the earthquake and could not be rebuilt in their original places; accordingly, these people were relocated to newly built towns or communities. Our participants were survivors who had moved from a village to a town or new community.

The participants were recruited through referrals from local physicians and posters displayed throughout the affected communities. The initial screenings for PTSD were conducted by using the PTSD Checklist-Civilian Version (PCL-C), which is a reliable and valid assessment of PTSD [23]. A PCL-C score greater than 45 was considered to be indicative of potential positive symptoms of PTSD. Then, a diagnosis was made by consensus between two experienced attending psychiatrists by using the Structured Clinical Interview based on the *Diagnostic and Statistical Manual of Mental Disorders*, 4th edition (DSM-IV) criteria for current PTSD, and the severity of the participants’ PTSD symptoms was determined by administering the Clinician-Administered PTSD Scale (CAPS) [24]. All participants met the DSM-IV criteria for current PTSD. Theoretical sampling was used to select the interviewees. Then, we decided which data to collect based on the initial analysis. The aim of the data collection was to develop a theory based on maximum diversity in terms of age, gender, education, socioeconomic position, employment status, symptom severity, and disaster experiences. In this study, the sample size was determined by reaching theoretical saturation, at which point the researchers concluded that the collected data were repeating, and no new insight could be reached.

### Data collection

In-depth semi-structured interviews were used for the data collection. A face-to-face, semi-structured interview was conducted with each participant at the local community medical center from 2016 to 2017. The participants were interviewed after the PTSD diagnosis was made by psychiatrists. The interviews were conducted by the first author. The first author had previously worked as a psychologist at a hospital and had been involved in another qualitative research project. Each interview was individually organized but usually began with open-ended questions, such as the following: Tell me about what happened to you after the earthquake. What did you feel? What did it mean to you? What are the changes to different aspects of your life? What are the changes in the different community? How has relocation affected your life? Complementary probing questions were added as needed that could relate to prior experiences with the disaster. The questions were developed based on previous interview data, and an ongoing analysis influenced the subsequent questions and the selection of the following participants. The interviews lasted between 30 and 60 min. The location of the interviews was quiet and private to protect the confidentiality and privacy of the participants.

### Data management and analysis

We followed the procedures for data management and analysis recommended by grounded theory [25, 26]. All interviews were transcribed verbatim from digital audio files. The in-depth interview data were independently analyzed by two researchers by using a grounded theory methodological analysis. The researchers regularly discussed the codes and categories. Based on grounded theory, the data collection and analysis were intertwined and recursive. Initially, we read through the transcripts to label the items in the data with codes that were inductively identified in the form of meaningful units by the two researchers who worked independently. This process is called open coding. The following step involved axial coding in which the codes were categorized through constant comparisons of the relationship between the major and minor themes in terms of similar and dissimilar meanings and actions. Additionally, selective coding, which refers to ongoing reflective analytical selection to form a larger theoretical framework by reintegrating and reorganizing the categories, was used to help explain the relationships between the categories. The credibility of this research was established through the long-term involvement of and close interaction with the participants, and field notes and memos were recorded from the beginning of the research and used as reference materials during the coding process. Faculty members who were not a part of the research team also supervised the strategies during the research process.

### Ethical considerations

This study was approved by the Clinical Research Ethics Committee of Southwest Hospital. The aim and process of the study were explained verbally and in writing to the participants, and the participants signed a written permission of informed consent. The participants were informed that they could decline participation at any time during the study. Confidentiality was maintained throughout the data collection, interview recordings, and process of presenting the results. Our current study was based on a previous qualitative study that aimed to understand suffering and emotion in Chinese earthquake survivors. We followed this previous study to focus exclusively on relocated earthquake survivors.

## Results

**Fig. 1:**
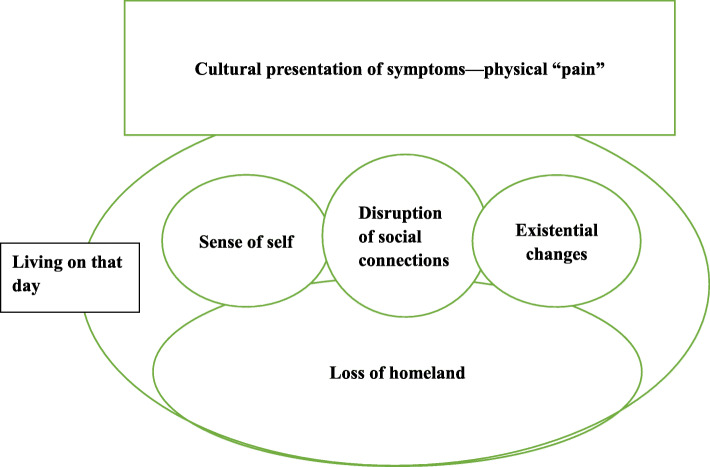
Loss of homeland—a theoretical model for understanding PTSD survivors’ traumatic experience

The emerging theory was inductively derived through the process of a constant comparative analysis. Figure 1 depicts a schematic representation of the categories and illustrates their interrelationships. In our model, a fundamental category called “loss of homeland” was discovered. Loss of homeland was the most important condition that influenced the relocated participants’ self-identity, social connections, and meaning system. These categories were grouped into existential changes, lost connections, and changes in identity. The disaster destroyed the participants familial, communal, connective, and cultural safe havens and left them with only traumatic memories. The participants narrated this aspect as though they were living on the day of the disaster; they feel that they always live on this day—in this moment. The participants cannot find a safe haven where they can recover and feel safe. The participants formed culturally bonded symptoms. Of the participants, ten were men, and thirteen were women. Of these participants, twelve were farmers, and eleven were farm workers. Nine participants lost loved ones in the quake; eighteen participants were married, two participants were divorced and three participants were widowed. Other detailed demographic information of the participants is summarized in Table 1.

**Table 1:** Relocated Participants’ Demographics (*N* = 23)

Participants		*N* = 23 (%)
Gender	Male	10 (43.4)
	Female	13 (56.6)
Mean age (years)		M = 42.2; SD = 10.1
CAPS	All score	M = 110.6; SD = 27.1
HAMA		M = 12.0; SD = 5.2
HAMD		M = 13.4; SD = 4.8
Marital status	Married	18 (78.3)
	Divorced	2 (8.7)
	Widowed	3 (13.0)
Education	Primary school	8 (34.8)
	Middle school	7 (30.4)
	High school	8 (34.8)
Occupation	Farmer	12 (52.2)
	Farmer worker	11 (47.8)
Loss of family members	No	14 (60.9)
	Yes	9 (39.1)

*P* Participant; *Edu* Year of Education; *CAPS* Clinician-Administered Posttraumatic Stress Disorder Scale; *HRMD* Hamilton Rating Scale for Depression; *HRMA* Hamilton Rating Scale for Anxiety

### Loss of homeland

Most relocated earthquake survivors lost their homeland due to the catastrophic earthquake. All participants presented the pain of a “loss of homeland”. The homeland is imbued with a range of meanings, feelings, experiences and a sense of belonging. The participants started their families in their homeland. The participants felt secure and protected in their homeland. One participant said the following:

Before the earthquake, I had a house with enough household appliances. Well, I also had land, and I could raise some pigs. This could help me make some money. After the earthquake, I had no house, no land. How could I make money? So, I had to do some part-time jobs to make a living. In the past, I had a house, and our family lived in the center of a big yard. I felt I was competent. After the earthquake, I became homeless, and I felt I was nobody all of a sudden. (p4, male, between 50 to 60 years old).

House, land, and home define the participants’ identity and happiness. The devastating earthquake caused tremendous loss and suffering.

I don’t even dare to look at my ruined house. I feel so sad every time I look at it. My house collapsed. Sometimes, I recall where my living room and kitchen were… Now, they are all gone. The yard was over there, you know. I don’t want to look at it. Why would you want to take a look if you have no house there? If you look at it, you will recall where you put noodles and other things. In the earthquake, the pigs and cows were killed. I can find next to nothing in the house, and I feel so down. (p7, female, between 40 to 50 years old).

Building a good home represents an enormous accomplishment. In turn, the loss of a home means the loss of identity. In China, home and person are considered an organic whole and cannot be separated. However, those who lost both their home and people suffered even more pain.

I only found a fat pig of mine in the farmland. I don’t know how it survived. I had a house, and all my family lived in it. All of a sudden, my wife and my wife’s sister were buried in there. You know, the most painful part is that nearly all my family died in the earthquake. They are all gone. (p16, male, between 50 to 60 years old).

In their homeland, there were people who the participants loved and cared for, and they established a family in the community. The loss of their homeland represents not only the loss of their home but also the loss of their loved ones, family members, relatives, friends, acquaintances, and the people with whom they grew up.

Well—how can I say it?—I feel very lonely now. We only have six family members, and four of them died in that earthquake. This hurt me the most. You see, I have no sons and grandsons to accompany me now, and I feel so lonely in the house. Sometimes, I take the plow to do some farm work, and when I look around, no one is there around me. Basically, I have no house and no family members now. I was the head of my village, and we had more than one thousand people in the village before the earthquake. Only five hundred of them survived. In the past, I could have dinner with every family when I collected the agriculture tax in their house. But, now, the house is gone, and the people are gone. I even stopped walking there. (p9, male, between 60 to 70 years old).

Sometimes, the loss means not only the loss of the participants’ home and family members but also the loss of their community. Their entire communities define the participants’ self-identity and existence, and deep emotions bind them within the community.

I told myself I need go to the mountain (the place where he lived before the earthquake) to take a look. I sat there and cried for a while. The whole community was gone; the village was gone. (p10, male, between 40 to 50 years old).

Another participant reported the following:

I did not like the new community. I got used to living in the mountains, and I felt good when I saw the mountains, rivers, and plants in my previous villages. I felt everything is strange to me in the new community. (p15, male, between 50 to 60 years old).

To a certain extent, a continuous longing for their hometown and home exists deep in the participants’ hearts. They understand that it is impossible to return to their hometown and their home again because the disaster destroyed everything. The participants are thus living with the pain of the loss of homeland.

### Existential changes

The wreckage left by the earthquake continues to evoke images of the disaster, loss, suffering, and death. The participants’ life experiences after the disaster make them ponder the existential problem, i.e., the meaning of life. The experience of suffering a catastrophic disaster can shatter one’s assumptive reality and sense of certainty in such a fundamental manner that survivors must not only cope with this loss but also rebuild their understanding of life. All participants presented changes in existential thinking after the disaster.

I have a life as long as my sons are alive. That’s what I mean. I take everything lightly now—unlike before, when different villages were fighting among themselves about the field boundaries. The problem does not exist anymore. As long as you are alive, what is the point of fighting for trivial things? Life is short. After I survived the earthquake, I adopted a different attitude toward many things in my life. (p7, female, between 40 to50 years old).

After experiencing a life or death situation, the participants began questioning the most important things in their lives.

I think I am more mature when viewing many things in my life. I think people should really treat friends well. After the earthquake, some of my friends died, and some left. We gradually lost contact with each other. I really adopt a more open attitude toward my life. In the past, I was not willing to spend money on eating and clothing. But, my attitudes have changed now. A person should eat and dress well in his or her life when he or she is alive. Nearly all my relatives have left, and I think I should try my best to help and keep in touch with the relatives who are still around me. (p5, female, between 20 to 30 years old).

The participants attached more importance to interpersonal relationships and family relationships. After experiencing this pain, they begin to redefine their lives and themselves.

What makes me happy is very simple right now. My sons frequently visit me. I can make some money, and my grandsons have fun every day. That’s what makes me happy… this is what I want. (p14, male, between 50 to 60 years old).

Many participants worked outside the community before the earthquake. Some participants moved back to their home to find a job nearby and live with their family members after the earthquake.

The kids have grown up, and I want family members to be together all the time. I am this type of person. I feel better and important when we are all together. (p19, male, between 40 to 50 years old).

Some participants claimed to experience existential suffering that prompted them to answer the following question: What is the life that I want to live? They formed new understandings of life and meaning.

### Lost social connections

Many participants claimed that most of their social connections were shattered after the earthquake. First, some participants said that the people with whom they lived had died in the earthquake or migrated to other places after the disaster. Second, most participants had to migrate to a new community, and they feel a loss of connection with the people and community with which they were familiar. Third, after the disaster, the participants became hypersensitive in their relationships.

I cannot find a person to talk to. I moved to this village after the earthquake. My good friends had moved away. Sometimes, it is so hard—so hard to find a friend to pour out my bitterness to. (p23, female, between 20 to 30 years old).

Many participants had to move to and settle in a new place. Losing contact with their friends was a consequence of the earthquake. In addition, the participants complained about experiencing oversensitivity in their social interactions.

In the new community, I feel it is so hard to communicate with other people… I feel everything is not right. For example, when you make a call and your voice is really loud, I will come to you and ask what happened or ask whether something terrible happened. I will feel so down if you don’t tell me. After several times, I stop keeping in touch with them, and I don’t know why I became like this after the earthquake. (p11, female, between 30 to 40 years old).

After the earthquake, the participants became oversensitive to negative information and information from people with whom they are not familiar. As a result, they began to forge a distorted conception of themselves and their interpersonal relationships.

It feels like you and me. We are two strangers, and we feel good about each other, and we want to be friends. However, there is a voice inside telling you that you cannot do this because that person may hurt you (just like the people did in the earthquake). All relationships have changed. You are alone in an isolated land without any resources. (p21, female, between 30 to 40 years old)

Due to this hypersensitivity towards interpersonal relationships and sense of inferiority after the earthquake, the participants proactively isolated themselves from other people. They stopped contacting other people simply out of self-protection.

I was afraid that the local people (people in the new community) would give me a weird look. After so many years, I walked away and lived alone after I had this feeling. My life circle is not as broad as before, and I don’t want to meet people. I spend more time in the house. (p22, male, between 40 to 50 years old).

The participants tend toward self-protection or avoidance of interpersonal conflict in the new community, and they choose to isolate themselves, which adds to their suffering through a loss of connections.

### Problem with the sense of self

For many of our participants, their sense of self was threatened by the disaster. Most participants reported that their sense of self was removed from the world that they actually inhabit. Many participants claimed to have a lower self-image and even self-alienation.

I feel I do not know who I am. The disaster really hurt me… I am not sure how the damn earthquake made me become a person like this: nervous, sensitive, suspicious… I also wonder whether I am crazy. Sometimes, I ask myself how you turn into a person like this—whether I am a human being or a ghost or neither. (p3, female, between 40 to 50 years old).

The men’s sense of self was threatened, and they negatively responded to information from their surroundings and other people. Most participants claimed to experience major changes in their self-perception after the earthquake.

I rarely look at myself in the mirror. Sometimes, when I look at myself, I feel so strange about myself in the mirror… and I also feel that I am inferior compared to other people. You have nothing that can compare with others. You are old. You cannot change anything… You cannot rebuild a house anymore. (p20, female, between 40 to 50 years old).

Another man also claimed that he felt differently about himself. He felt that he had turned into a different person. He was also confused about why and how his personality changed. The integrity of self had been threatened and transformed by the traumatic experiences. The integrity of self was also deeply connected to their land, tradition, and community.

### Living on that day

“Living on that day” implies a day-to-day life full of traumatic experiences and memories. It is a different type of life from the participants’ past, normal life experiences. The participants experience intrusive memory, fear, phobia, helplessness, and nightmares day and night. The participants attempt to live a life as normal as possible but struggle with these terrifying feelings. They become easily irritated, hyper-vigilant, and out of control and are preoccupied by the feeling of loss and hopelessness. All of these experiences are explained by the fact that they are in continuous pain.

I just cannot help recalling the whole scene. When I think about those terrible memories, I feel like I am having a mental breakdown. To be honest, everyone will feel scared in that situation. We are fortunate people because we did not die in that earthquake. What I feel terrified about is not the collapse of those houses but the death of so many people I knew. I am in my forties, and I just cannot help remembering those memories. (p6, male, between 40 to 50 years old).

Many emotions, such as guilt and regret about the earthquake, do not fade over time. These emotions still linger and corrode their minds.

On that day, I invited my classmate’s son to help me carry the corn in from the land. My classmate’s son was killed by stones on his way to my house. Forty to fifty people died in my village, which is a huge stress for me. I cannot stop thinking about them. When I think, I just cry. (p13, female, between 40 to 50 years old).

Regardless of their attempts, the memories of that day constantly intrude upon their everyday lives.

I have so many negative thoughts, even when I see an acquaintance and I can remember how he treated me in the earthquake. I feel very reluctant to have these thoughts, but they just come up automatically. (p21, female, between 30 to 40 years old).

Nightmares followed. Many participants reported that all their dreams are related to that day.

You did not see that scene, and I cried for a long time after I came back. Sometimes, I dreamed about those kids who were dug out from stones without hands and heads. Blood was everywhere. Some kids were not dead when they were found, but their hands and arms were gone, and blood was rushing. It felt so sad when you saw that. I do not want to live when I recall those scenes. (p2, female, between 30 to 40 years old).

It has been 8 years since the earthquake occurred, but the participants still live in the shadow of the earthquake and the pain that it caused. The participants are similar to a bird that frightens at the sight of a bow.

I run immediately whenever there is a small aftershock. Sometimes, I think about whether I need to find a place to put some water and food for an emergency in case an earthquake happens. I think all the time about what I should do if there is an earthquake again. When some aftershocks hit, I just run, even if it is a really small quake. (p8, female, between 40 to 50 years old)

“Living on that day” involves the survivors’ intrusively and uncontrollably recalling images of the disaster or pain caused by the earthquake.

### Culturally bonded symptoms: physical pain

Physical uneasiness is a major complaint of survivors with PTSD. Survivors with PTSD believe that everything is related to their physical problem [27]. All participants consistently claimed experiencing some physical disturbances or somatization symptoms rather than describing their psychosocial pain. Describing physical disturbances is a way to describe psychosocial pain among Chinese people and a way to seek help and social support.

I had been under the stones for an hour in the earthquake. My chest became numb… After the earthquake, I always feel uncomfortable, and I feel faint all the time. My memory also became bad. I want to get something sometimes, and when I walk there, I remember nothing. (p10, male, between 40 to 50 years old).

Poor memory is a major complaint of all participants. Many participants attribute their poor memory to a physical disease, and they visited a hospital for evaluation. The symptoms disrupt their daily functions.

I find I have a problem when I talk with people now. When I want to talk about something, I say other things. When I think of one name in my mind, I say a different name… Do you think my brain has some disease? There is another problem. I feel faint immediately when I am under pressure. In recent years, people have given me money a couple of times, and I count how much. If they ask me how much money, I just cannot remember. (p12, female, between 30 to 40 years old).

Another participant expressed similar concerns as follows:

Sometimes, I make a call, and after I dial the number, I do not remember who I called. I have to wait for the person to speak, and I can then remember who I called. I have had this problem several times recently. (p18, male, between 40 to 50 years old).

Chinese people mainly describe their physical symptoms rather than mental problems or feelings. They experience memory and physical symptoms in their everyday lives, which causes many disturbances.

## Discussion

In our model, we can observe the dynamic interplay among relocation, psychosocial vulnerability and the cultural expression of disease, which demands a new framework for understanding PTSD. Typically, the disaster impacted the relocated participants’ families, social relationships, communities, cultural integrity, beliefs and life stories. The significance of land for Chinese people has generally been ignored in the Western literature. However, because of its central role in identity construction, connection, and, in particular, a sense of belonging, the concept of land is important for understanding Chinese life experiences and feelings.

### Land is the foundation of Chinese society

Based on our model, we can observe that the homeland forms the foundation of safety, belonging, meaning, connection, identity, and integrity of the self and body. Attachment to land is a characteristic of rural society. In rural China, it is normal for farmers to settle in one place for generations; it would be abnormal for them to migrate. The sense of belonging to interpersonal relationships, the sense of self, and the well-being of individuals, family, and community are experienced and occur on the land. Relocation and the loss of homeland lead to problems with the sense of self, social connections, and meaning. Previous researchers have also claimed that people who perceive the environment as stable, consistent, predictable, manageable, and meaningful are more likely to have a sense of coherence and develop a better ability to cope with traumatic incidents and vice versa [28]. Without a stable psychosocial, cultural, and community-holding environment, Chinese victims feel that they still live on the day of the earthquake, which threatens their ontological security. Based on the suffering that they experienced, they form their “symptoms”, which are largely culturally bounded. The microcultures that shape individuals’ illness experiences are largely negative and disregarded in mainstream psychiatry.

### Survivors rebuild their interpersonal connections, community, culture, and meaning

In our study, the participants’ experiences of loss of connections helped identify the impacts of traditional cultural values on Chinese patients’ experiences of suffering. Chinese society emphasizes interpersonal interrelatedness, which has become an important part of Chinese people’s personality [29, 30]. Given the primacy of interpersonal relationships in contemporary Chinese culture in general, the topic of interpersonal connections has become a central concern among Chinese people. Hsiao and his colleagues note that Chinese people are relational, i.e., their personal identity is embedded in social relationships [31]. Another study also supports that future disaster mental health services must help earthquake survivors build effective social ties and recover their social support systems because social support after an earthquake is a very important factor in preventing post-disaster mental health problems in China [32, 33].

In the phenomenological research, the feeling that one becomes another person is an indicator of how one’s self-image and identity change after a disaster. Due to the loss of their homeland, community, and social connections, victims feel that they have become totally different people compared to their past selves, which contributes to avoidance, vigilance, and feelings of vulnerability. Furthermore, increased feelings of avoidance, vigilance, and vulnerability have been described as symptoms of PTSD. Previous research that simply describes the symptoms of PTSD may fail to understand victims’ experiences of the dis-integrity of self, which is the origin and core of their symptoms. Consistent with the literature, after a traumatic incident in life, without a stable holding environment, the integrity of self is usually threatened, which presents as low self-esteem and increased self-doubt [34, 35].

As claimed by many relocated survivors, disaster is a powerful threat to the life of earthquake victims, and regular safety and certainty are not taken for granted. Traumatic events shatter one’s community and stable surroundings in which the individual’s meaning is embedded, which leads to a sense of meaninglessness, hopelessness, impotence, uncertainty, and lack of control in life. As mentioned by many existential psychologists and scholars, the threat evokes an existential exploration of what the most important things are in a person’s life and what he or she wants to live for [36, 37].

Based on the participants’ narratives, traumatic memory is always “unforgotten” and relived in their everyday experiences. The biological foundation of the traumatic symptoms of PTSD have been well documented in different experiential, psychophysiological, and neurobiological studies. In addition to the biological understanding of the disease, based on our sociocultural model, we can clearly observe that without a safe and stable harbor, i.e., community, connections, cultural resources, etc., victims live in an unpredictable, unsafe, and overwhelming setting. They have no way to recover from the disaster. Without helping them rebuild connections, community, culture, and meaning, we simply label their symptoms, which does not help them recover from their trauma.

### Somatic symptoms tell a story of deep social, cultural, and spiritual suffering

Somatic changes and physical health complaints were often displayed, as bodily stories were experienced more by illustrating than by speaking. Chinese people do not verbally express their feelings or personal emotions. The major complaints of physical health changes rather than psychological disturbances indicate a confirmation of the personal history of their experiences. The close interrelatedness between psychic and physical states in the Chinese culture may suggest that unspeakable pain could facilitate a deeper cultural understanding of disaster survivors’ experiences [38]. The meaning of physical discomfort and somatic presentation in the functions of eating, sleeping, memorizing, paying attention, dreaming, breathing, acting, working, and remembering implies that disasters affect every aspect of everyday life and shatter victims’ inner and outer harmony. Listening to these earthquake survivors’ sorrowful narratives and exploring their everyday experiences in depth provide a key to understanding the cultural meaning of PTSD symptoms. It is pivotal for clinicians to listen to and understand victims’ culturally specific metaphorical expressions of trauma as culture-specific expressions strongly reflect sociocultural and historical contexts and indicate potential approaches to the cultural adaptation of clinical interventions [39].

#### Conclusion

Past quantitative research has provided one aspect of their experiences. Our current qualitative research forms another picture of the life experiences of relocated earthquake survivors with PTSD and can help in devising methods to boost survivors’ resilience by promoting social support and recovery in a resilient community. With better information regarding the dynamic interactions by which post-disaster relocation affects mental health, mental health professionals and policy makers can make more informed decisions in terms of disaster relocation policy and manage post-disaster psychological disturbances by focusing on both places and people.

#### Practical and clinical implications

First, future mental health professionals and policy makers must understand how place relates to mental health, which is important for delivering effective “contextually sensitive” interventions and policies. Second, mental health professionals or policy makers can use the findings from this study to inform the development of comprehensive “land”-based programming that aims to increase the sense of belonging, recover community routines, build a sense of identity, decrease social exclusion, and promote inclusion in new communities. Third, the embodied experiences of somatization should be explored and considered to understand earthquake survivors’ inner suffering and pain. Future mental health services could focus on patients’ somatic complaints, which may reflect emotional distress, as the failure to incorporate somatic metaphors and Chinese conceptualizations of distress may lead to a failure in understanding posttraumatic psychological disturbances [40].

#### Limitations

Despite this study’s contributions, it has the following limitations. First, although we followed the theoretical sampling of grounded theory, our study group of participants who have experienced relocation to other communities represents a small share of the millions relocated due to the Sichuan earthquake; therefore, the theoretical soundness of this study is limited. Second, this study involved participants from several moderately to severely damaged communities, and most patients had low socioeconomic backgrounds. Therefore, our results may not be generalizable to populations with a higher socioeconomic background or from mildly damaged communities. Third, some patients suffered complicated grief and prolonged PTSD, and how relocation impacted their complicated grief and prolonged PTSD was not addressed in the current study.

## Conclusions

Chinese people’s self-identity is closely related to land, community, and social ties. The severe disaster shattered not only the homeland of the earthquake survivors but also their sense of belonging, security and self-identity associated with land, community and social relationships. Mental health services need to address the cultural factors that might shape survivors’ vulnerability to traumatic incidents. Future clinical workers must find a natural way to help earthquake survivors narrate their traumatic experiences in a more acceptable and indigenous manner, such as rituals, ceremonies, and traditional coping strategies to integrate unintegrated, fragmented, and fragile episodic memories into a semantic memory system to form a comprehensive and sequential autobiographical memory of their family, community culture, and homeland.

## Data Availability

The datasets obtained and/or analyzed during the current study are not publicly available due to the confidentiality of the study but can be obtained from the corresponding author upon reasonable request.

## Abbreviations

PCL-C: PTSD checklist-civilian version
PTSD: Posttraumatic stress disorder DSM-IV: Diagnostic and Statistical Manual of Mental Disorders, 4th edition
